# Crossing the blood–brain barrier with carbon dots: uptake mechanism and *in vivo* cargo delivery†

**DOI:** 10.1039/d1na00145k

**Published:** 2021-05-31

**Authors:** Elif S. Seven, Yasin B. Seven, Yiqun Zhou, Sijan Poudel-Sharma, Juan J. Diaz-Rucco, Emel Kirbas Cilingir, Gordon S. Mitchell, J. David Van Dyken, Roger M. Leblanc

**Affiliations:** Department of Chemistry, University of Miami 1301 Memorial Dr. Coral Gables FL 33146 USA rml@miami.edu; Department of Physical Therapy, University of Florida 101 Newell Dr. Gainesville FL 32603 USA; McKnight Brain Institute, University of Florida 1149 Newell Dr. Gainesville FL 32610 USA; Department of Biology, University of Miami 1301 Memorial Dr. Coral Gables FL 33146 USA

## Abstract

The blood–brain barrier (BBB) is a major obstacle for drug delivery to the central nervous system (CNS) such that most therapeutics lack efficacy against brain tumors or neurological disorders due to their inability to cross the BBB. Therefore, developing new drug delivery platforms to facilitate drug transport to the CNS and understanding their mechanism of transport are crucial for the efficacy of therapeutics. Here, we report (i) carbon dots prepared from glucose and conjugated to fluorescein (GluCD-F) cross the BBB in zebrafish and rats without the need of an additional targeting ligand and (ii) uptake mechanism of GluCDs is glucose transporter-dependent in budding yeast. Glucose transporter-negative strain of yeast showed undetectable GluCD accumulation unlike the glucose transporter-positive yeast, suggesting glucose-transporter-dependent GluCD uptake. We tested GluCDs' ability to cross the BBB using both zebrafish and rat models. Following the injection to the heart, wild-type zebrafish showed GluCD-F accumulation in the central canal consistent with the transport of GluCD-F across the BBB. In rats, following intravenous administration, GluCD-F was observed in the CNS. GluCD-F was localized in the gray matter (*e.g.* ventral horn, dorsal horn, and middle grey) of the cervical spinal cord consistent with neuronal accumulation. Therefore, neuron targeting GluCDs hold tremendous potential as a drug delivery platform in neurodegenerative disease, traumatic injury, and malignancies of the CNS.

## Abstract 2

Glucose-based carbon dots (GluCDs) can cross blood–brain barrier in zebrafish and rat after intravenous injections and accumulate in neurons in rat CNS. Cell uptake of GluCDs involve glucose transporter proteins in a budding yeast model.

## Graphical abstract

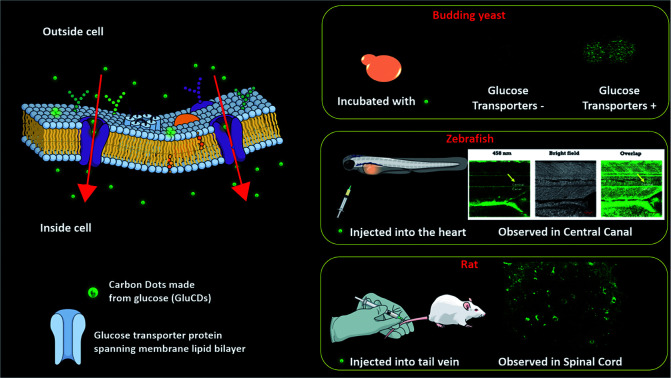

## 1. Introduction

The blood–brain barrier (BBB) is the single most important factor limiting the development of neurotherapeutics for neurological disorders and tumors.^1,2^ The BBB only allows passage of certain nutrients such as glucose, amino acids, and neurotransmitter precursors from the blood to the brain and blocks other molecules present in the bloodstream. As a result, more than 98% of the small molecule drugs and 100% of large molecule drugs cannot cross the BBB.^1^ Therefore, the variety of the therapeutic agents available for central nervous system-related diseases and cancers is very limited.^3^ A drug delivery system (DDS) that can carry therapeutic agents across the BBB is highly sought after. In recent years, nanoparticle (NP)-mediated DDS have received wide attention for the BBB penetration.^3–8^ In most cases, these NPs rely on ligands such as apolipoprotein or transferrin to pass through the BBB *via* receptor-mediated endocytosis.^9,10^ However, when NP-based platforms need a bulky protein such as transferrin, drug loading and targeting efficiency decreases because of its resulting steric hinderance and avidity stemming from the bulky ligand on the surface of the NP.^9^ Here, we developed *self-targeting* carbon dots (CDs) that can cross the BBB *in vivo* without the need of additional ligands.

As a new class of carbon-based nanomaterials, CDs have quickly drawn attention since their discovery.^11^ CDs have been widely synthesized, characterized, and applied as promising nanocarriers for drug delivery in the past 20 years.^12^ Many unique properties of CDs that are not often observed in other carbon-based nanomaterials such as small size (1–10 nm), photoluminescence (PL) and abundant surface functionalities have been reported in several studies.^12–14^ In addition, CDs display high water dispersibility, good biocompatibility, and nontoxicity.^15^ Moreover, CDs' surfaces can also easily be decorated with molecules such as fluorophores and drugs.^16^ Considering these merits, CDs are very promising drug nanocarriers for future drug delivery.^6,17^ However, far less is known regarding the biological interactions and cell uptake mechanisms of CDs *in vitro* and *in vivo*. Understanding the uptake mechanism of CDs is crucial for development of drug delivery systems. To the best of our knowledge, no mechanistic cell uptake studies of CDs has been published and the BBB crossing mechanism is not well-understood. Furthermore, despite several studies report *in vivo* models of the CD-based drug delivery systems for cancers and bioimaging, the number of studies targeting central nervous system (CNS) is very sparse.^18,19^ In many cases, BBB crossing studies lack mechanistic approaches and therefore are suggestive.^20^ Therefore, developing new CD-based drug delivery platforms to cross the BBB holds significant promise and understanding their mechanism of transport are highly novel and very important.

Cell surface receptors and transport proteins comprise a pathway to internalize extracellular molecules, often by recognizing their surface functional groups. Because CDs have residues of their precursor molecules on their surfaces,^20,21^ the ligand residues likely have high affinity to the receptors of their precursors. Glucose transporter protein 1 (GLUT1) has been shown to be involved in BBB crossing of some NPs such as liposomes or micelles, which require further surface modifications to attach glucose.^22–24^ However, the size of the surface-modified liposomes or micelles, ∼50–150 nm, is very large for an efficient transport by GLUT1 and is likely the cause of low level of accumulation in the brain tissue. In contrast, CDs are desirable because of their ultrasmall size (<10 nm) and that they can incorporate precursor-resembling moieties on their surfaces intrinsically. Accordingly, we hypothesize that (1) CDs derived from glucose (GluCDs) have surface functional groups similar to glucose, (2) the GluCD cellular uptake requires glucose transporter proteins, and (3) GluCDs cross the BBB *in vivo*. To test these hypotheses, we synthesized GluCDs and their conjugate to a fluorescent marker (fluorescein; GluCD-F), studied the cell uptake mechanism of GluCDs in a budding yeast model, assessed the ability of GluCDs to cross the BBB and carry a small molecule cargo to the CNS in zebrafish and rat models and determined the GluCD-F distribution in the rat CNS.

**Fig. 1:**
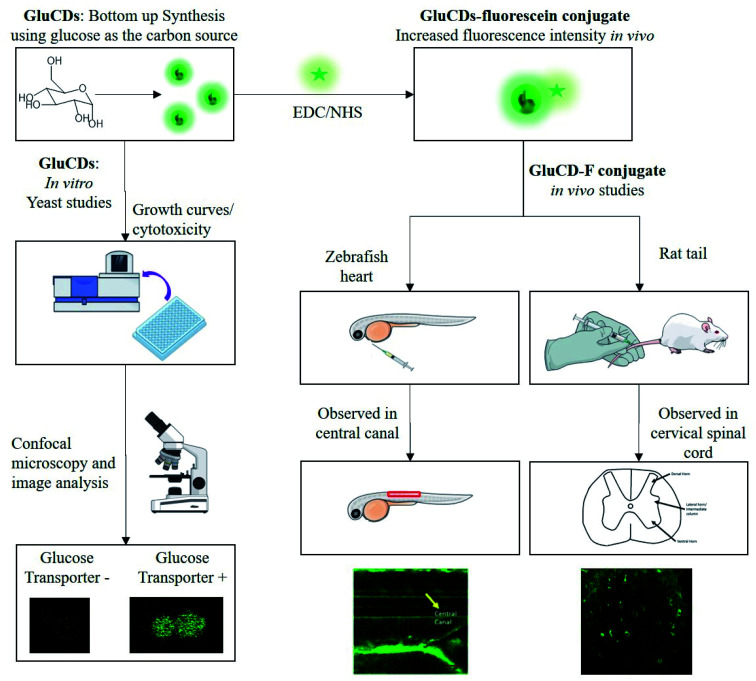
Flowchart of the methods for *in vitro* and *in vivo* studies. GluCDs were used for *in vitro* studies and GluCD-F was used for *in vivo* studies.

Here, we report our novel findings that: (1) GluCDs cross the BBB in zebrafish and rat models without the need of conjugation to a targeting ligand for receptor-mediated transport. (2) GluCDs can transport cargo to CNS. (3) The uptake mechanism of GluCDs requires glucose transporter proteins in a yeast model. The methodology of biological studies is summarized in Fig. 1.

## 2. Materials and methods

### 2.1. Preparation and characterization of GluCDs

GluCDs were prepared with d-glucose (VWR International, LLC; Radnor, PA) as the only precursor using the same method we previously reported.^25^ Briefly, 20 ml of 0.3 M d-glucose solution was prepared in DI-water obtained from Direct-Q 3 water purification system (EMD Millipore Corp.; Chicago, IL) with a resistivity of 18 MΩ cm at 20.0 ± 0.5 °C and surface tension of 71.2 mN m^−1^ at the same temperature. The solution was heated up to 200 °C in 30 min and kept at 200 °C for 5 h in a Teflon-coated autoclave reactor (DW-5MB2-BG61, BAOSHISHAN, China) using a muffle furnace (BF51800 Series; Thermo Scientific; Rockford, IL). The reaction mixture was left to cool down to room temperature followed by centrifugation for 20 min at 9000 rpm. Then, the supernatant was taken, filtered through a syringe filter with 0.2 μm pore size (VWR International, LLC; Radnor, PA), and adjusted to neutral pH using super saturated NaOH solution. The sample was dialyzed against DI-water using a 1 kDa MWCO dialysis membrane (Spectra/Por®; VWR International, LLC; Radnor, PA) for three days changing the water every 12–24 hours. Sample was lyophilized to yield solid product using a FreeZone 4.5 L cascade benchtop freeze dry system (Labconco, Co.; Kansas City, MO). All chemicals were used as received without further purification.

GluCDs were characterized using ultraviolet-visible (UV/Vis), fluorescence and Fourier transform infrared (FTIR) spectroscopies as well as using transmission electron microscopy (TEM). The FTIR spectroscopy was performed with attenuated total reflection (ATR) accessories.

Cary 100 UV/Vis spectrophotometer (Agilent Technologies; Wilmington, DE) and Horiba Jobin Yvon Fluorolog-3 spectrofluorometer (Horiba Scientific; Edison, NJ) with a slit width of 5 nm for both excitation and emission were utilized to obtain UV/Vis absorption and photoluminescence (PL) emission spectra of GluCDs and GluCD-F conjugate dispersed in DI-H_2_O, respectively. For UV/Vis absorption and PL emission spectra measurements, quartz spectrophotometer cells and quartz fluorometer cell with 1 cm pathlength (Starna Cells, Inc.; Atascadero, CA) were used, respectively. FTIR spectra of the lyophilized d-glucose, GluCDs, and GluCD-F with air as background were obtained using FTIR-ATR spectrometer (PerkinElmer Inc; Waltham, MA).

For morphological studies and determination of mean particle size of GluCDs, TEM images were collected with a JEOL 1200X transmission electron microscope (JEOL USA, Inc.; Peabody, MA). To prepare the sample for TEM imaging, GluCDs were well dispersed in DI-water and sonicated to avoid agglomeration using a Branson 1510 ultrasonic cleaner (Gaithersburg, MD). The aqueous dispersion was then deposited on a carbon-coated copper grid and air dried before the TEM screening. The diameters of GluCDs were measured using ImageJ image processing software for three times. The mean diameter was reported with standard error of the mean, and the size distribution histogram was plotted using Origin 9.1 (OriginLab Corp., USA).

### 2.2. Preparation of GluCD-fluorescein (GluCD-F) conjugate

GluCDs were covalently conjugated to 5-(aminomethyl)-fluorescein hydrochloride (Thermo Fisher Scientific, Life Sciences; Carlsbad, CA) *via* 1-ethyl-3-(3-dimethylaminopropyl) carbodiimide (EDC) and *N*-hydroxy succinimide (NHS) coupling chemistry (Millipore-Sigma; St. Louis, MO). First, the –COOH groups on GluCDs were activated by EDC and NHS. 30 minutes later, 5-(aminomethyl)-fluorescein was added to the mixture. The mixture was stirred overnight protected from light. At the end, –COOH group on GluCDs' surface and –NH_2_ of the 5-(aminomethyl)-fluorescein form a stable amide bond. GluCDs conjugated to 5-aminomethyl-fluorescein (GluCD-F) was purified by dialysis against DI-water, allowing unreacted small molecules to escape from the dialysis membrane with 1 kDa MWCO. Fluorescence emission of the dialysis water was measured periodically to monitor the purification and to ensure no free fluorescein is remained mixed with the GluCD-F conjugate. Finally, the purified conjugate was lyophilized to yield powdered product. Successful conjugation was confirmed by UV/Vis, fluorescence, and FTIR-ATR spectroscopies.

### 2.3. Quantum yield (QY) calculations

The fluorescence quantum yield (QY, *Φ*) was calculated using the equation below^26^:*Φ*_CDs_ = *Φ*_ST_((*A*_ST_*F*_CDs_)/(*A*_CDs_*F*_ST_))(*η*_CDs_*η*_ST_)^2^Subscripts ST and CDs stand for the standard reference and carbon dots, respectively. *A* refers to the absorbance, *F* is the integrated area under the PL emission curve, and *η* is the refractive index of the solvent used for the measurements. Quinine sulphate (*Φ* = 54%) in 0.1 M H_2_SO_4_ (*η* = 1.33)^27^ and lucigenin (*Φ* = 67%)^28^ in DI-water were used as two independent standards for QY calculations of GluCDs dispersed in DI-water (*η* = 1.33). Absorbance of GluCDs was measured at a concentration in which the absorption peak was kept under 0.05 at 350 nm and the same concentration was used for PL emission measurements. QY of GluCDs was calculated separately for each standard.

### 2.4. *Saccharomyces cerevisiae* studies

#### 2.4.1. Culture preparation

Two strains with and without glucose transporters with close genetic background were selected to use in this study. Barcode (BC) strain, which expresses all glucose transporter proteins, was derived from BY 4709.^29^ EBY.VW 5000 strain was provided as a generous gift by Eckhard Boles. EBY.VW 5000 is a hexose-transport-negative strain (*hxt* null), in which all members of *HEX* genes have been deleted.^30^ EBY.VW 5000 cells were grown in YPM (1% yeast extract, 2% peptone, 2% maltose) medium whereas BC cells were grown in YPD (1% yeast extract, 2% peptone, 2% glucose) medium.^29,30^ Three replicate cultures were grown overnight. The cultures were diluted in fresh media and incubated with GluCDs dispersed in phosphate-buffered saline (PBS) with a final concentration of 0.2 mg CDs per ml and a density of 2 × 10^8^ cells per ml. In control cultures, same amount of PBS was added to the cells as vehicle. The cultures were incubated at 30 °C in glass tubes rotating for 4 hours. The cultures were grown to log phase to ensure that the glucose transporters were expressed. Table 1 summarizes name of the strains used, glucose transporter expression and treatment.

**Table 1:** Yeast strains, glucose transporter expression and treatment for each group

Group	Glucose transporter expression	Treatment
BC-C	+	Vehicle (PBS)
BC-T	+	GluCDs
EBY.VW 5000-C	−	Vehicle (PBS)
EBY.VW 5000-T	−	GluCDs

#### 2.4.2. Cytotoxicity (growth curve)

The saturated cultures of both strains were washed with PBS twice and diluted 10^3^ times in a 96-well microplate using Biomek Automation system (Beckman Coulter; Indianapolis, IN). GluCDs dispersed in PBS were then added to the yeast cells at a final concentration of 0.2 mg ml^−1^. In control wells only PBS was added. The cells were then grown at 30 °C with constant orbital shaking at 280.8 rpm while absorption at 660 nm (OD_660_) was measured every 15 minutes for 45 hours in a plate reader (Tecan Nanoquant Infinite M200 Pro, Switzerland) to plot the growth curves. Growth curves were then fitted to the standard logistic equation using a custom MATLAB code (Mathworks 2018) to calculate the growth rates. Student's *t*-test was used to compare the growth rates of control groups and treatment groups of the same strain, and *p* < 0.05 was considered as significant.

##### Confocal microscopy

After the cells reached the log phase, the cultures were washed twice with PBS, resuspended in 4% paraformaldehyde (PFA) solution, incubated for 10 minutes to fix the cells followed by PBS wash twice and resuspended in PBS. 20 μl of the homogenized cell-suspension was used to prepare a wet mount. The control slides were also processed in the same way. The slides were then immediately viewed under confocal microscope.

At least 200 cells per sample were counted while collecting the fluorescence data. Confocal images were acquired using a Leica SP5 confocal microscope (Leica Microsystems Inc., Buffalo Grove, IL) using a bright field channel and a fluorescent channel with the excitation wavelength of 458 nm. Same settings were used for each group.

#### 2.4.3. Image analysis

ImageJ was used to quantify the fluorescence intensities to evaluate nanoparticle uptake. First, bright field images of yeast cells were median filtered to calculate an overall background value of each image. Background value was subtracted from its respective bright field image. The region of interests (ROIs) in each bright field image were identified by binarizing the images using an adaptive threshold value determined by a fixed percentile value of fluorescence intensities of each image. Then, a weighted-fluorescence intensity was calculated for each image as follows:
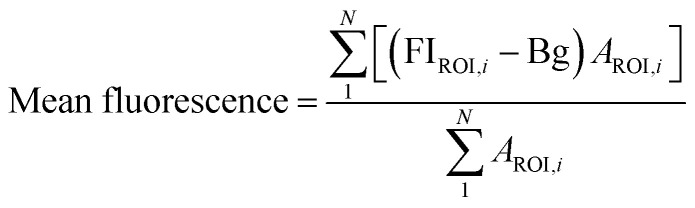
FI_ROI_ is the average fluorescence intensity from a given ROI, Bg is the background fluorescence intensity, *A*_ROI_ is the area of a given ROI. Mean fluorescence intensity was calculated for all control and treatment groups.

Since yeast cells have autofluorescence independent from the image background, we present our data with (raw) and without (delta) autofluorescence. The delta fluorescence intensity for BC-T and EBY.VW 5000-T was calculated using the equation below for subtraction of the autofluorescence:
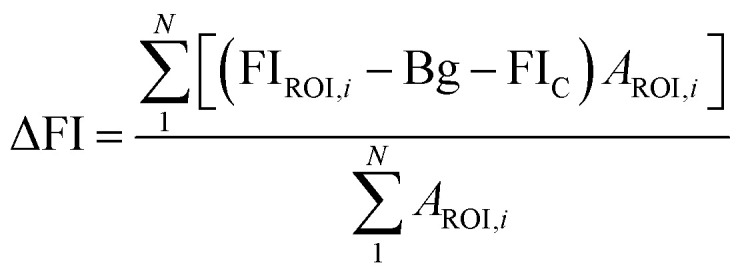
ΔFI is the delta fluorescence intensity and FI_C_ is the mean fluorescence intensity of control group (*i.e.* autofluorescence) calculated using the first equation. JMP Pro 14.0 software was used for statistical analyses. Student's *t*-test was used and *p* < 0.05 was considered as significant. Data was presented as mean ± standard error of the mean. Bivariate analysis was used to assess the correlation between the area of ROI and fluorescence intensity.

### 2.5. Zebrafish injection and bioimaging

Wild-type zebrafish (*Danio rerio*) at 5 days post fertilization were anesthetized using tricaine. The aqueous dispersion of GluCD-F (10 mg ml^−1^) was intravascularly injected into the heart of anesthetized zebrafish and soon after, the treated zebrafish were observed using a Leica SP5 confocal microscope under bright field and fluorescence (excitation at 458 nm) channels. The animal care protocol for all procedures used in these experiments complies with the guidelines of National Science Foundation and was approved by the University of Miami Animal Care and Use Committee. Wild-type zebrafish were obtained from the Zebrafish Core Facility at University of Miami.

### 2.6. Rat studies

Adult male Sprague-Dawley rats (Envigo; Indianapolis, IN) weighing 400 g were used to test if GluCD-F cross the BBB. Experimental protocols were approved by the University of Florida Institutional Animal Care and Use Committee and were conducted in accordance with the Animal Welfare Act, the Public Health Service Policy on Humane Care and Use of Laboratory Animals, and the NIH Guide for the Care and Use of Laboratory Animals (2011). Rats had access to food and water *ad libitum* and were kept at 12 h light–dark cycle. Intravenous injections and terminal procedures were performed under anesthesia. Anesthesia was induced (3%) and maintained (2.5%) with isoflurane. Depth of anesthesia was monitored by the absence of toe pinch and palpebral reflexes.

For intravenous injections, rats were placed on a heated surgical table. The skin surface around the lateral tail vein injection site was rubbed 3 times alternating chlorhexidine with alcohol. Following tail vein catheterization (24 Gauge; Surflash, Somerset, NJ), rats were administered with GluCD-F in sterile saline or vehicle. Once the catheter was removed, the injection site was gently pressurized for ∼3 minutes. Then, anesthesia was discontinued. Rats were monitored regularly and kept awake for 4 h. There was no abnormal sign following the injection. Then, rats were sacrificed by transcardial perfusion using 0.01 M PBS at 4 °C followed by 4% paraformaldehyde in 0.01 M PBS (pH 7.4). Brain and spinal cord were harvested, post-fixed in 4% paraformaldehyde in 0.01 M PBS (4 °C, pH 7.4) overnight and cryoprotected in 20% followed by 30% sucrose solution (4 °C). All the incubation and storage steps after harvesting were done under protection from light (foiled whenever possible). Cervical spinal cord and medulla were sectioned to 40 μm thick slices in the transverse plane using a freezing microtome (SM2010R, Leica; Buffalo Grove, IL). Sectioned slices were kept in antifreeze solution (30% glycerol + 30% ethylene glycol in 0.1 M PBS) at −20 °C. Three slices per spinal segment and 8 medulla slices were uniformly sampled to verify the consistency of labeling and mounted on charged slides with hard-set anti-fade media (Vector Labs; Burlingame, CA). No fluorescent immunohistochemistry was performed to prevent non-specific antigen binding-induced fluorescence. Slides were imaged at 20× magnification with a fluorescence microscope (BZ-X710; Keyence Co.; Osaka, Japan) with a GFP filter (BZ-X, model no.: OP-87763). The same exposure times were used across groups.

## 3. Results and discussion

### 3.1. Synthesis and characterization of glucose-based carbon dots and fluorescein conjugate

**Fig. 2:**
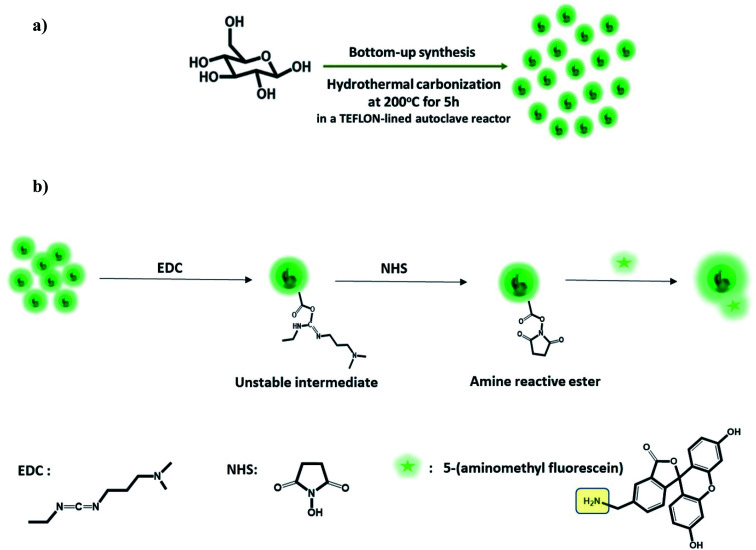
(a) Schematic representation of the bottom-up carbon dot synthesis *via* hydrothermal carbonization of d-glucose in a TEFLON-lined autoclave reactor at 200 °C for 5 h (GluCDs). (b) Schematic representation of the conjugation of 5-amionomethyl-fluorescein to glucose carbon dots (GluCD-F) using EDC/NHS coupling reaction. Structures of EDC, NHS and 5-aminomethyl-fluorescein is given on the bottom of the figure.

GluCDs were synthesized using the same methodology reported in our previous work.^25^Fig. 2a shows a schematic representation of the synthesis of GluCDs with glucose as the sole precursor *via* a hydrothermal bottom-up method and used for *in vitro* studies. The QY of GluCDs was obtained as 0.1% using two standard references, namely quinine sulfate and lucigenin. Since the QY of the GluCDs is not high enough for *in vivo* bioimaging studies, GluCDs were conjugated to 5-aminomethyl-fluorescein for zebrafish and rat studies for better imaging. The fluorescein conjugation also serves as a proof-of-principle model to test if GluCDs can carry a small molecule drug or cargo across the BBB as a potential drug delivery platform. Fig. 2b is a schematic representation of the conjugation of GluCDs to 5-(aminomethyl)-fluorescein *via* EDC/NHS amidation coupling. As GluCDs have carboxylic acid surface functional groups^25^ and 5-(aminomethyl)-fluorescein has a primary amine group, they form a stable amide bond through the EDC/NHS conjugation reaction.

**Fig. 3:**
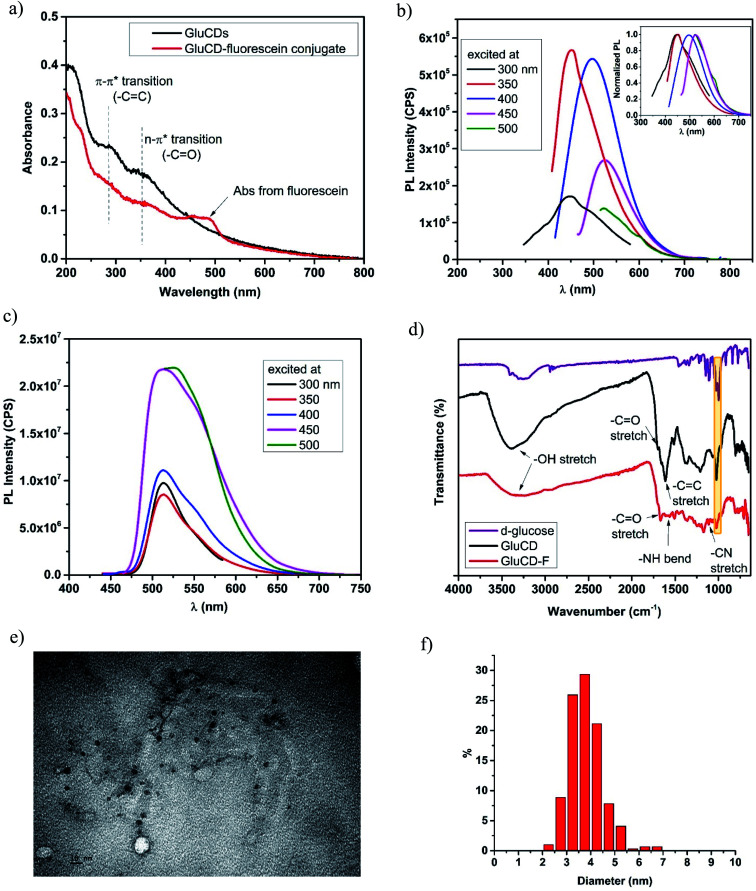
Characterization of GluCDs and GluCD-F. (a) UV/Vis spectra of GluCDs and GluCD-F, (b) PL emission spectrum of GluCDs show excitation dependent PL emission. The maximum emission peak was at 450 nm when excited at 350 nm. Inset shows the normalized PL emission. (c) PL emission spectrum of GluCD-F. The PL emission of the conjugate is excitation independent. The maximum emission peak was at 526 nm when excited at 500 nm. (d) FTIR-ATR spectra of d-glucose (purple), GluCDs (black) and GluCD-F conjugate (red). (e) TEM image of GluCDs. Scale bar represents 10 nm. (f) Particle size distribution histogram of GluCDs.

GluCDs were characterized by UV/Vis, fluorescence, and FTIR spectroscopies as well as TEM microscopy. Fig. 3a shows the UV/Vis spectra of GluCDs (black) and GluCD-F (red). The band around 260–325 nm can be attributed to the π–π* transition (C=C) whereas the band around 325–400 nm can be attributed to the n–π* transition (C=O).^25^ The peak in the GluCD-F's absorption spectrum at around 492 nm stems from fluorescein. Fig. 3b and c show the PL emission spectra of GluCDs and GluCD-F, respectively. The PL emission of GluCDs is excitation wavelength dependent shifting to red with increasing excitation wavelength. The PL emission maximum is at 450 nm when excited at 350 nm. The inset of Fig. 3b is the normalized PL emission showing the excitation dependent red shift of PL emission from 445 to 525 nm. Fig. 3c shows the PL emission spectrum of the GluCD-F. As opposed to the PL emission of GluCDs, the PL emission of GluCD-F is not dependent on the excitation wavelength. The fluorescence intensity of fluorescein in the conjugate is 10^2^ fold higher than that of GluCDs at the same concentration as the fluorescence QY of fluorescein is 92% in water.^31^ The emission peak of GluCD-F is dominantly from fluorescein, which explains the independence from the excitation wavelength and the significant increase in the intensity. It must be noted that any fluorescence from an organic fluorophore is independent of the excitation wavelength. The emission maximum of the conjugate is at 526 nm when excited at 500 nm showing a slight red shift compared to the fluorescein's emission maximum at 520 nm.^32^ This slight red shift may be due to extending the π-conjugation system. To ensure there is no free fluorescein in the GluCD-F dispersion, we dialyzed the conjugate against DI-H_2_O for three days using a 1 kDa MWCO dialysis membrane changing the water every 12–24 hours. We took samples from dialysis water each time we changed it and measured the fluorescence emission of the dialysis water. At the end of three days, the PL emission intensity detected in dialysis water was negligible (Fig. S-1 in the ESI†). The decrease in the fluorescence intensity of dialysis water shown in Fig. S-1† supports that there is no free fluorescein mixed with the GluCD-F. Therefore, the conjugation between GluCDs and fluorescein was successful, and the absorption peak in Fig. 3a (red) and emission peak in Fig. 3c stem from the fluorescein conjugated to GluCDs.

To further characterize GluCDs and confirm the successful conjugation, FTIR spectra in the solid state were obtained using an ATR accessory with air as background. Fig. 3d shows FTIR spectra of the precursor (d-glucose) (purple), GluCDs (black) and GluCD-F (red). Both spectra of GluCDs and GluCD-F are different from the d-glucose's spectrum, especially between 2000–1500 cm^−1^. All three samples have an –OH stretch at around 3200–3400 cm^−1^. GluCDs' spectrum shows a strong –OH stretch which can be attributed to the hydroxyl and carboxyl functional groups. GluCD-F's –OH/–NH stretch is broader and weaker compared to GluCDs. GluCDs' spectrum shows –C=O stretch at around 1705 cm^−1^ consistent with the formation of –COOH groups. GluCDs-F's spectrum is different from GluCDs' spectrum especially in the carbonyl and amide region between 1800–1500 cm^−1^. –C=O stretch in GluCD-F's spectrum is slightly shifted to the right compared to that in the spectrum of GluCDs. The bands between 1660 and 1550 cm^−1^ can be attributed to amide I from carbonyl stretch and amide II from –N–H bond. The formation of a new band at 1105 cm^−1^ is consistent with –C–N stretch. Formation of new bands suggests a successful formation of the amide bond in the GluCD-F. Sharp peak at 1024 cm^−1^ in both GluCDs and GluCD-F (shown in yellow box, Fig. 3d) is consistent with the C–O–C stretch of the anhydroglucose ring supporting that GluCDs and GluCD-F have surface moieties similar to glucose.

Next, TEM images were used to determine the morphology of GluCDs in *X*–*Y* plane, calculate the average size, and determine the size distribution (Fig. 3e and f). Fig. 3e shows circular particles consistent with spherical structure of CDs. ImageJ software was used to measure the diameters of GluCDs. 277 particles were counted and the average diameter size of GluCDs was found to be 3.77 ± 0.17 nm. GluCDs have a very narrow diameter distribution (95% confidence interval: 2.58 to 5.42 nm).

### 3.2. GluCD uptake requires glucose-transporters in budding yeast *S. Cerevisiae*

The vast majority of organisms share the need to uptake and metabolize glucose at the cellular level.^33^ Thus, glucose transporters are highly conserved across species from yeast to zebrafish, and rodents.^34^ For mechanistic studies, we used budding yeast, *Saccharomyces cerevisiae*, as the model organism. Since Hxt permeases are homologues of GLUT in the yeast,^35^ we utilized wild-type and *hxt* null *S. cerevisiae*.

We chose two *S. cerevisiae* strains with similar genetic background to study the effect of glucose transporters in the cell uptake mechanism of GluCDs. EBY.VW 5000 is a mutant strain that exhibits the deletion of all glucose transporters and barcode (BC) strain, which is derived from BY4709, is a strain expressing all glucose transporters. EBY.VW 5000-T and BC-T refer to the treatment groups which were incubated with 0.2 mg ml^−1^ GluCDs dispersed in PBS for 4 hours before confocal imaging whereas EBY.VW 5000-C and BC-C refer to the control groups which were treated the same way with treatment groups except that control groups were incubated in PBS instead of GluCDs dispersion.

**Fig. 4:**
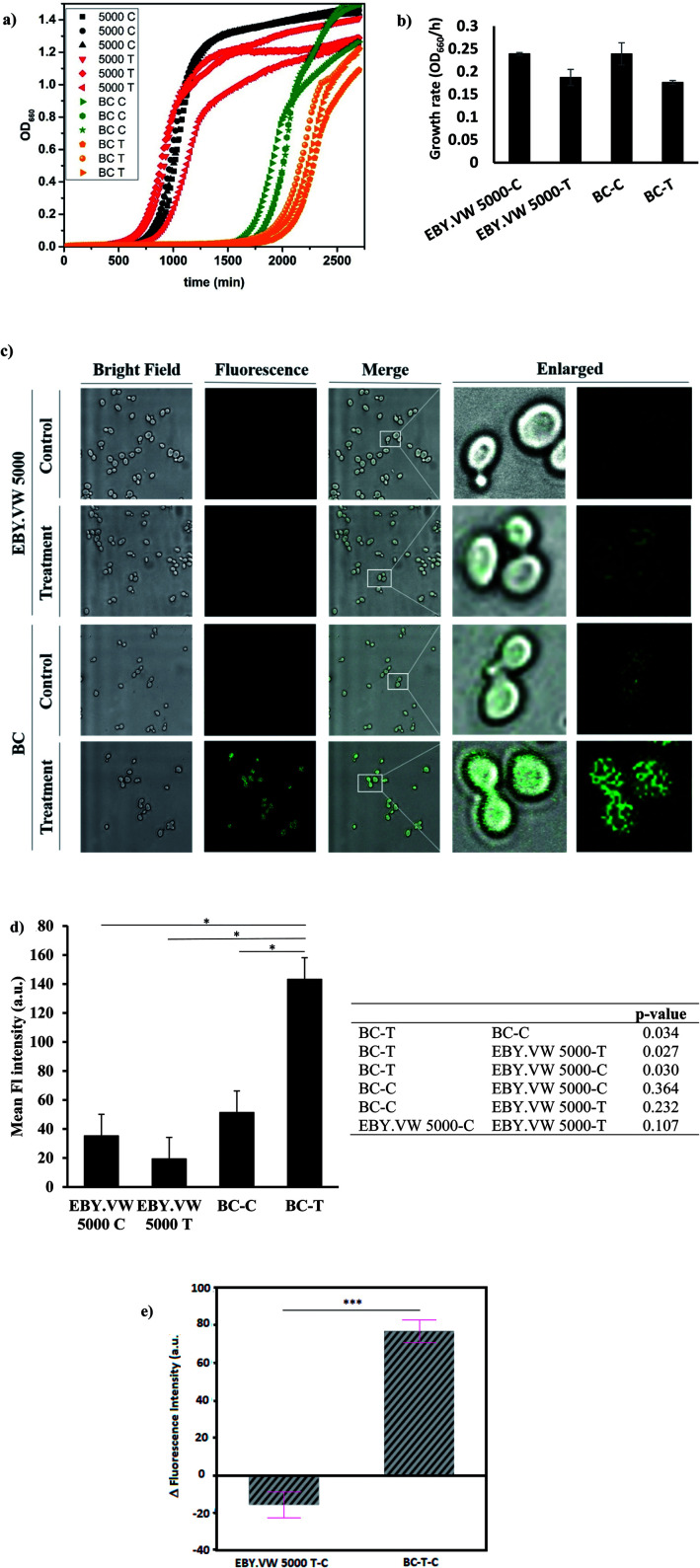
(a) Growth curves of all groups in three replicates: black curves are EBY.VW 5000-C, red curves are EBY.VW 5000-T, green curves are BC-C and orange curves are BC-T (b) bar graph shows the mean growth rates (OD_660_/h) of EBY.VW 5000-C, EBY.VW 5000-T, BC-C and BC-T with standard error of the mean. Growth rates of treatment groups show no significant difference compared to respective controls (*p* > 0.05), (c) representative confocal images of EBY.VW 5000 and BC strains' control and treatment groups under bright field and fluorescence channels, merged images as well as enlarged images. Only BC-T shows bright fluorescence when treated with GluCDs, (d) bar graph shows the mean fluorescence intensity of all groups based on the quantitative fluorescence intensity analysis using confocal images. The table shows *p*-value for comparisons between each group. Fluorescence intensity of the treatment group of BC (Hex+) strain (BC-T) is significantly higher than that of all other groups (*p < 0.05) and there is no significant difference in the fluorescence intensity between other groups. (e) Delta fluorescence intensity of treatment groups of BC-T-C (Hex+) and EBY.VW 5000-T-C (Hex−) strains after subtracting the corresponding autofluorescence, ***p value < 0.0005.

First, we tested the toxicity of GluCDs on yeast cells. For cytotoxicity studies, the yeast cells in three independent cultures for both EBY.VW 5000-T and BC-T were incubated in a 96-well plate with 0.2 mg ml^−1^ GluCDs at 30 °C for about two days measuring the absorbance at 660 nm every 15 min. Three individual cultures for each strain EBY.VW 5000-C and BC-C were grown as control in the same plate. Fig. 4a shows the growth curves for all groups: control and treatment for both strains with and without glucose transporters, with three replicates each. The growth rates (OD_660_/hour) of both EBY.VW 5000 and BC strains showed no significant difference between the treatment and control groups (*p*-values for EBY.VW 5000 and BC are 0.11 and 0.13, respectively) supporting that incubation with GluCDs has no significant toxicity on the yeast cells that may cause slower growth or death (Fig. 4b). The lag phase of BC strain was longer than that of EBY.VW 5000. However, this difference was observed for all replicates in both control and treatment groups. Thus, it is less likely to stem from GluCDs treatment.

Our central hypothesis was that glucose transporter proteins are involved in the cell uptake of GluCDs and therefore there will be significantly higher fluorescence intensity in the BC-T cells as a result of GluCDs accumulation compared to that of the EBY.VW 5000-T cells. Fig. 4c shows confocal images of yeast cells with and without glucose transporters which were treated with GluCDs or vehicle (PBS). Only BC-T (Hex+, treated with GluCDs) showed notable fluorescence. While other groups showed some fluorescence, they were not bright. To analyze the fluorescence intensity of the confocal images, first we compared control and treatment groups (Fig. 4d). There was a significant difference in terms of the fluorescence intensity between the control and treatment groups of the BC strain which expresses all glucose transporters (*p* = 0.034), suggesting that increase in fluorescence in BC-T stems from the GluCDs accumulation in the cells. There was no significant difference in fluorescence intensity between the control and treatment groups of glucose transporter negative strain EBY.VW 5000 (*p* = 0.11; Fig. 4d). To test the central hypothesis, we compared EBY.VW-T and BC-T groups. The mean fluorescence of GluCD-treated BC-T cells (Hxt+) was significantly higher than that of GluCD-treated EBY.VW 5000 cells (Hxt−; *p* = 0.027; Fig. 4d) supporting our hypothesis that glucose transporter proteins play a crucial role in the cell uptake of GluCDs. To further compare the two treatment groups (Hex+ and Hex−), we subtracted the autofluorescence as autofluorescence intensity based on native fluorophores such as tryptophan, pyridoxine and flavins may differ between different strains.^36^ The emission stemming from tryptophan and pyridoxine is in the blue region with maxima at 325 and 385 nm when excited at 290 and 340 nm, respectively.^37^ Therefore, considering that we collected the confocal images upon the excitation of 400 nm, it is highly unlikely to have autofluorescence from tryptophan or pyridoxine. However, the emission from riboflavin with a maximum at 535 nm^37^ when excited at 460 nm overlaps with the emission of GluCDs. Therefore, to eliminate any bias based on a possible difference between the autofluorescence of EBY.VW 5000 and BC strains, we subtracted the mean fluorescence intensity of control groups (autofluorescence) from their corresponding treatment groups. This is referred as delta fluorescence intensity. The comparison between the delta fluorescence intensities of EBY.VW 5000-T-C and BC-T-C also resulted in a significant difference (*p* = 0.0005) (Fig. 4e). It should be noted that the autofluorescence of EBY.VW 5000-C and BC-C were not significantly different (*p* = 0.492) possibly because they are from similar genetic background. When the autofluorescence of each untreated group was subtracted from its respective treatment group, the difference between the BC-T-C and EBY.VW 5000 T-C became clearer, further lowering *p*-value. Nevertheless, the results with or without subtraction of the autofluorescence consistently show a significant difference supporting glucose-transporter dependent cell uptake of GluCDs.

To further confirm that the significant difference between the mean fluorescence intensity of yeast strains with and without glucose transporters were not artificial, we extended the analysis to include the correlation between the area of ROIs and fluorescence intensity. The ROIs may contain several clustered yeast cells. Therefore, there is a possibility that there are empty spaces between clusters of cells counted in the ROI area which lacks the fluorescence signal. Consequently, the larger area of ROIs may falsely decrease the mean fluorescence intensity. If there is correlation between the ROI size and fluorescence intensity, the results may be biased. To test this, first we plotted the area distributions for all groups (Fig. S-2 and S-3†), calculated the mean ROI areas (Table S-1†) and compared the difference between the ROI areas of each group (Table S-2†). Results showed a significant difference between ROI areas of EBY.VW 5000 and BC for both control and treatment groups. The ROI areas of EBY.VW 5000 control and treatment are larger than that of BC control and treatment. However, there is no significant difference between the controls and treatments of the same strain. To test the correlation between ROI area and fluorescence intensity, we used bivariate analysis (Fig. S-4†). Results showed that the fluorescence intensity increases with increasing area for both strains (Fig. S-4†), and the correlation between the area of ROIs and fluorescence intensity was very weak for both strains (*R*^2^ = 0.01 for EBY.VW 5000 and *R*^2^: 0.0003 for BC). So, bias based on the correlation between fluorescence intensity and ROI area is quite weak and in the opposite direction of the assumption that the larger area may falsely decrease the mean fluorescence. This analysis further supports that the fluorescence in the treated BC strain stems from the accumulated GluCDs, thus the glucose transporters are crucial for the cell uptake of GluCDs.

### 3.3. GluCD crosses the BBB in zebrafish *Danio rerio* following intravascular delivery

**Fig. 5:**
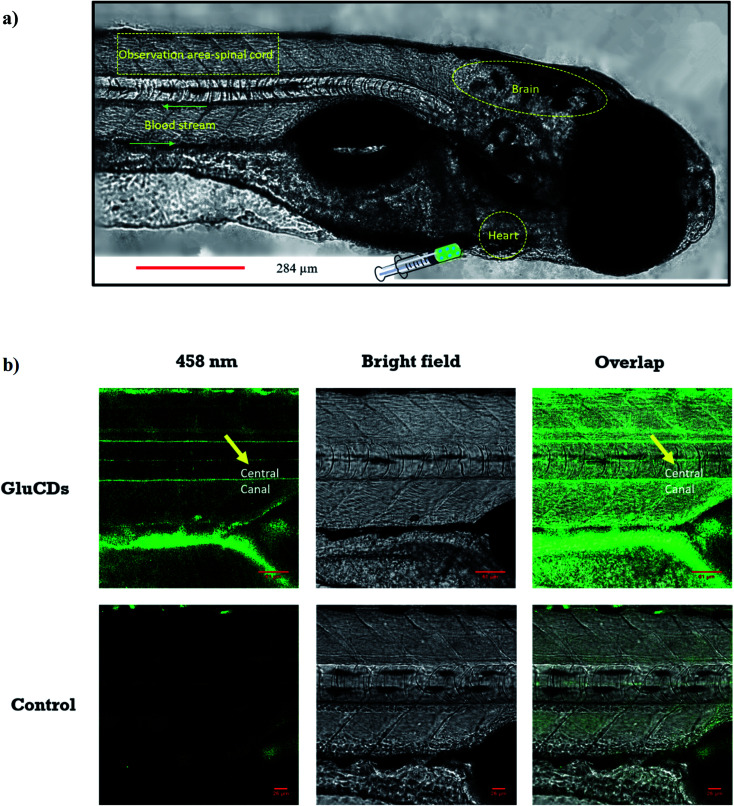
(a) Confocal images of wild-type zebrafish showing the injection route, heart, blood stream, CNS and observation area (central canal of spinal cord). (b) Accumulation of GluCDs-F in the CNS of zebrafish. The yellow arrow indicates the central canal of spinal cord of zebrafish.

First, we assessed the ability of GluCDs to cross the BBB using the wild-type zebrafish *Danio rerio* as an *in vivo* model. Zebrafish offers a significant advantage for whole body fluorescence imaging studies as they have a nearly transparent body at 5^th^ day post fertilization when all major organs and systems are fully functional.^38,39^ GluCDs were conjugated to 5-(aminomethyl)-fluorescein hydrochloride to increase the PL intensity for confocal bioimaging. To examine whether the CDs could cross the BBB, 10 mg ml^−1^ GluCD-F conjugate's aqueous dispersion was injected into the heart of zebrafish (*n* = 12). The administration site and observation area is shown in Fig. 5a. Under a confocal microscope with the excitation at 458 nm, green fluorescence was observed in the central canal of spinal cord of zebrafish (Fig. 5b). On the contrary, the control group did not show fluorescence. In one of our previous studies, the CDs prepared from carbon nanopowder, sulfuric acid, and nitric acid could not cross the BBB without the conjugation to transferrin.^40^ In the same study, we reported that in the absence of transferrin, CD-fluorescein conjugate alone could not cross the BBB showing that fluorescein is not able to carry the CDs across the BBB. Here, on the contrary, we showed that GluCDs could overcome the BBB without the need of transferrin conjugation. However, since the PL intensity of bare GluCDs is too low to be detected *in vivo* using the confocal microscopy, we used the GluCD-F for *in vivo* bioimaging. This result also supports the hypothesis that GluCDs are not only able to cross the BBB but also can succesfully carry fluorescein from the bloodstream to the CNS when conjugated covalently, which shows that GluCDs are promising drug delivery platforms to carry small molecules across the BBB. Furthermore, we chose the dye fluorescein as a model for small-molecule drugs because of its inability to cross the BBB like many small-molecule drugs. Fluorescein along with evans blue is widely used to assess the integrity of the BBB as these dyes are not able to cross the intact BBB whereas a significant staining in the brain can be observed if the BBB has been disrupted or opened osmotically.^41,42^

### 3.4. GluCD-F crosses the BBB and localizes to the grey matter in rats following intravenous delivery

Next, we tested if the GluCD-F could cross the BBB in rats. We used rat as a second *in vivo* model to confirm our zebrafish results in a mammalian species and analyze the accumulation and distribution of GluCD-F since rodent models have long been recognized for their genetic and physiological similarities to humans. Here, we determined GluCD-F localization at the brain areas involved in the neural control of breathing. Importantly, respiratory failure is a major cause of death in neurotraumatic, neurodegenerative, and neuromuscular disorders such as spinal cord injury and amyotrophic lateral sclerosis (ALS). Therefore, crossing BBB and targeting brainstem and cervical spinal cord (cSC) is of paramount importance since these brain regions contain critical core elements of the respiratory neuronal network.

**Fig. 6:**
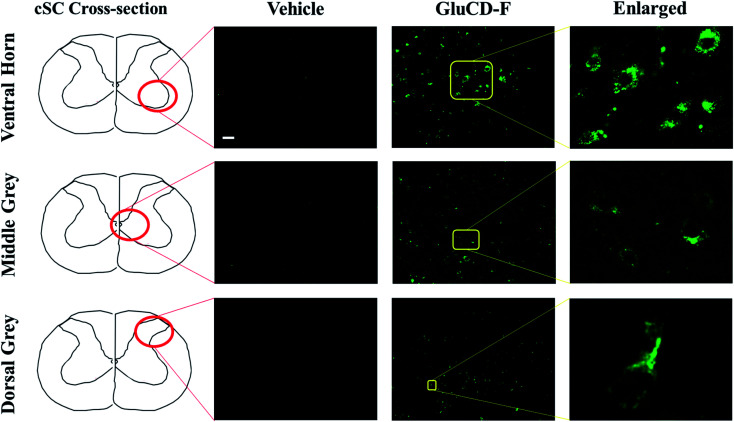
GluCD-F was observed in different regions of rat CNS. Sample images show GluCD-F localization in the ventral horn of the cervical spinal cord (top), middle grey matter of the cervical spinal cord (middle), and dorsal horn of cervical spinal cord (bottom). Corresponding cervical spinal cord cross-section camera obscura schematic showing the area of the image taken are displayed at the left column. Scale bar: 50 μm.

GluCD-F-treated rats exhibited normal behavior following injections compared to vehicle-treated rats. Four hours after intravenous injections, GluCD-F was observed in rat CNS (*n* = 5). Histological assessments indicated green fluorescence in brainstem (Fig. S-5†) and in the ventral horn, medial grey, and dorsal grey of the cSC (Fig. 6), while white matter appear to have very little fluorescence. In the grey matter, fluorescence was present within cell-types with large cell bodies and some projections. The size, shape, and location of these cell types were consistent with α-motor neurons, interneurons, and some dorsal horn neurons, suggesting that the GluCD-F accumulate predominantly in neurons.^43–46^ Furthermore, GluCD-F appear to be present as puncta in the *peri*-nuclear areas in neuronal somas. In dendrites, fluorescence was observed both as puncta and in diffuse pattern. The absence of fluorescence in white matter, where neuronal axons are present, suggests that axonal localization was limited if any. Although the same image acquisition settings were used in all groups and areas, we observed a slightly higher ‘background’ fluorescence in grey matter, which is likely due to diffuse, low-level dendritic fluorescence and fluorescence scattering in the tissue.^47^

Our results in rats are consistent with the zebrafish experiments such that GluCDs can pass the BBB and carry small cargo molecules. In particular, it is important to show transport through the BBB in a mammalian model, since this may have implications for potential drug delivery applications. It has been shown that the expression of glucose transporters GLUT3 (neurons) and GLUT1 (BBB) are upregulated, and the expression of GLUT3 has been increased as much as 300% 4–48 h after pathological events such as severe diffuse traumatic brain injury or hypoxic, ischemic and excitotoxic insult.^48–52^ Therefore, delivering drugs to the neurons after injury *via* GluCDs holds a great potential as a novel therapeutic strategy.

## 4. Conclusion

In summary, we report a versatile nanoparticle that can cross the BBB *via* glucose transporters and likely accumulate in neurons. The synthesis of GluCDs is green, relatively easy, labor and cost efficient. The size and biocompatibility of GluCDs make them a desirable nanoplatform for drug delivery. CDs were prepared using d-glucose as the only precursor and the cell uptake mechanism of GluCDs was studied using budding yeast as a model organism. The glucose transporter negative strain showed significantly less fluorescence from GluCDs, thus supporting that glucose transporters play a crucial role in the cell uptake mechanism of GluCDs. Then, zebrafish was used as an *in vivo* BBB model to test whether GluCDs could pass the BBB and accumulate in the CNS when they were intravascularly injected into the heart. To increase the fluorescence intensity for *in vivo* bioimaging and model the drug carrying potential, GluCDs were conjugated to fluorescein. Rats were used as the second *in vivo* BBB model to confirm the results of zebrafish experiments in mammalians and study the accumulation areas of GluCD-F. Results showed that GluCDs cannot only cross the BBB in various animal models but also can carry a dye, which also serves as a model for small molecule drugs, into the CNS. Our results bring insight into the promising potential of GluCDs as DDS for the CNS, particularly to neurons after injury.

## Conflicts of interest

Authors declare no conflict of interest.

## Supplementary Material

NA-003-D1NA00145K-s001

## References

[cit1] Pardridge W. M. (2005). NeuroRx.

[cit2] Ye D., Zimmermann T., Demina V., Sotnikov S., Ried C. L., Rahn H., Stapf M., Untucht C., Rohe M., Terstappen G. C., Wicke K., Mezler M., Manningac H., Meyer A. H. (2021). Nanoscale Adv..

[cit3] Deeken J. F., Löscher W. (2007). Clin. Cancer Res..

[cit4] Masserini M. (2013). ISRN Biochem..

[cit5] Mintz K. J., Mercado G., Zhou Y., Ji Y., Hettiarachchi S. D., Liyanage P. Y., Pandey R. R., Chusuei C. C., Dallman J., Leblanc R. M. (2019). Colloids Surf., B.

[cit6] Zhou Y., Liyanage P. Y., Devadoss D., Rios Guevara L. R., Cheng L., Graham R. M., Chand H. S., Al-Youbi A. O., Bashammakh A. S., El-Shahawi M. S., Leblanc R. M. (2019). Nanoscale.

[cit7] Liyanage P. Y., Zhou Y., Al-Youbi A. O., Bashammakh A. S., El-Shahawi M. S., Vanni S., Graham R. M., Leblanc R. M. (2020). Nanoscale.

[cit8] Zhang M., Bishop B. P., Thompson N. L., Hildahl K., Dang B., Mironchuk O., Chen N., Aoki R., Holmberg V. C., Nance E. (2019). Nanoscale Adv..

[cit9] Ceña V., Játiva P. (2018). Nanomedicine.

[cit10] Zhou Y., Peng Z., Seven E. S., Leblanc R. M. (2018). J. Controlled Release.

[cit11] Liu J., Li R., Yang B. (2020). ACS Cent. Sci..

[cit12] Xiao L., Sun H. (2018). Nanoscale Horiz..

[cit13] Kang Z., Lee S. T. (2019). Nanoscale.

[cit14] Mintz K. J., Zhou Y., Leblanc R. M. (2019). Nanoscale.

[cit15] Zhang Z., Yi G., Li P., Zhang X., Fan H., Zhang Y., Wang X., Zhang C. (2020). Nanoscale.

[cit16] Hettiarachchi S. D., Graham R. M., Mintz K. J., Zhou Y., Vanni S., Peng Z., Leblanc R. M. (2019). Nanoscale.

[cit17] Peng Z., Miyanji E. H., Zhou Y., Pardo J., Hettiarachchi S. D., Li S., Blackwelder P. L., Skromne I., Leblanc R. M. (2017). Nanoscale.

[cit18] Bhattacharya K., Mukherjee S. P., Gallud A., Burkert S. C., Bistarelli S., Bellucci S., Bottini M., Star A., Fadeel B. (2016). Nanomedicine.

[cit19] Baldrighi M., Trusel M., Tonini R., Giordani S. (2016). Front. Neurosci..

[cit20] Zheng M., Ruan S., Liu S., Sun T., Qu D., Zhao H., Xie Z., Gao H., Jing X., Sun Z. (2015). ACS Nano.

[cit21] Phan L. M. T., Gul A. R., Le T. N., Kim M. W., Kailasa S. K., Oh K. T., Park T. J. (2019). Biomater. Sci..

[cit22] Qin Y., Fan W., Chen H., Yao N., Tang W., Tang J., Yuan W., Kuai R., Zhang Z., Wu Y., He Q. (2010). J. Drug Targeting.

[cit23] Xie F., Yao N., Qin Y., Zhang Q., Chen H., Yuan M., Tang J., Li X., Fan W., Wu Y., Hai L., He Q. (2012). Int. J. Nanomed..

[cit24] Anraku Y., Kuwahara H., Fukusato Y., Mizoguchi A., Ishii T., Nitta K., Matsumoto Y., Toh K., Miyata K., Uchida S., Nishina K., Osada K., Itaka K., Nishiyama N., Mizusawa H., Yamasoba T., Yokota T., Kataoka K. (2017). Nat. Commun..

[cit25] Seven E. S., Sharma S. K., Meziane D., Zhou Y., Mintz K. J., Pandey R. R., Chusuei C. C., Leblanc R. M. (2019). Langmuir.

[cit26] Fery-Forgues S., Lavabre D. (1999). J. Chem. Educ..

[cit27] Moore D., Happe J. (1961). J. Phys. Chem..

[cit28] Halawa M. I., Wu F., Zafar M. N., Mostafa I. M., Abdussalam A., Han S., Xu G. (2020). J. Mater. Chem. B.

[cit29] Levy S. F., Blundell J. R., Venkataram S., Petrov D. A., Fisher D. S., Sherlock G. (2015). Nature.

[cit30] Wieczorke R., Krampe S., Weierstall T., Freidel K., Hollenberg C. P., Boles E. (1999). FEBS Lett..

[cit31] Magde D., Wong R., Seybold P. G. (2002). Photochem. Photobiol..

[cit32] Wang L., Wang Y., Ragauskas A. J. (2010). Anal. Bioanal. Chem..

[cit33] Thorens B., Mueckler M. (2010). Am. J. Physiol.: Endocrinol. Metab..

[cit34] Roy A., Dement A. D., Cho K. H., Kim J. H. (2015). PLoS One.

[cit35] Liu Z., Sanchez M. A., Jiang X., Boles E., Landfear S. M., Rosen B. P. (2006). Biochem. Biophys. Res. Commun..

[cit36] Bhatta H., Goldys E. M. (2008). FEMS Yeast Res..

[cit37] Maslanka R., Kwolek-Mirek M., Zadrag-Tecza R. (2018). J. Microbiol. Methods.

[cit38] Veldman M. B., Lin S. (2008). Pediatr. Res..

[cit39] Ali S., Champagne D. L., Spaink H. P., Richardson M. K. (2011). Birth Defects Res., Part C.

[cit40] Li S., Peng Z., Dallman J., Baker J., Othman A. M., Blackwelder P. L., Leblanc R. M. (2016). Colloids Surf., B.

[cit41] Baba M., Oishi R., Saeki K. (1988). Naunyn-Schmiedeberg's Arch. Pharmacol..

[cit42] Kozler P., Pokorný J. (2003). Physiol. Res..

[cit43] Watson C., Paxinos G. and Kayalioglu G., The spinal cord: a Christopher and Dana Reeve Foundation text and atlas, Academic Press, 2009

[cit44] Seven Y. B., Nichols N. L., Kelly M. N., Hobson O. R., Satriotomo I., Mitchell G. S. (2018). Exp. Neurol..

[cit45] Seven Y. B., Perim R. R., Hobson O. R., Simon A. K., Tadjalli A., Mitchell G. S. (2018). J. Physiol..

[cit46] Seven Y. B., Simon A. K., Sajjadi E., Zwick A., Satriotomo I., Mitchell G. S. (2020). Exp. Neurol..

[cit47] Moretti C., Gigan S. (2020). Nat. Photonics.

[cit48] Vannucci S. J., Seaman L. B., Vannucci R. C. (1996). J. Cereb. Blood Flow Metab..

[cit49] Hamlin G. P., Cernak I., Wixey J. A., Vink R. (2001). J. Neurotrauma.

[cit50] Zhang X. L., Li D. Y., Zhao F. Y., Qu Y., Mu D. Z. (2009). Sichuan Daxue Xuebao, Yixueban.

[cit51] Weisová P., Concannon C. G., Devocelle M., Prehn J. H., Ward M. W. (2009). J. Neurosci..

[cit52] Zovein A., Flowers-Ziegler J., Thamotharan S., Shin D., Sankar R., Nguyen K., Gambhir S., Devaskar S. U. (2004). Am. J. Physiol.: Regul., Integr. Comp. Physiol..

