# Supplementary material for: Crossing the blood–brain barrier with carbon dots: uptake mechanism and in vivo cargo delivery
Source: Nanoscale Adv. 2021 May 31;3(13):3942–53. doi: 10.1039/d1na00145k (PMC8243484; doi:10.1039/d1na00145k)
Supplement: NA-003-D1NA00145K-s001 [file NA-003-D1NA00145K-s001.pdf]

Electronic Supplementary Material (ESI) for Nanoscale Advances.
This journal is © The Royal Society of Chemistry 2021

Crossing the blood-brain barrier with carbon dots: uptake mechanism and *in vivo* cargo delivery

Elif S. Seven[1], Yasin B. Seven[2,3], Yiqun Zhou[1], Sijan Poudel-Sharma[4], Juan J. Diaz-Rucco[1], Emel Kirbas Cilingir[1], Gordon M. Mitchell[2,3], J. David Van Dyken[4], Roger M. Leblanc[1]*

[1] *Department of Chemistry, University of Miami, 1301 Memorial Dr., Coral Gables, FL, USA 33146*

[2] *Department of Physical Therapy, University of Florida, 101 Newell Dr, Gainesville, FL, USA 32603*

[3] *McKnight Brain Institute, University of Florida, 1149 Newell Dr, Gainesville, FL, USA 32610*

[4] *Department of Biology, University of Miami, 1301 Memorial Dr., Coral Gables, FL, USA 33146*

*Corresponding Author

rml@miami.edu

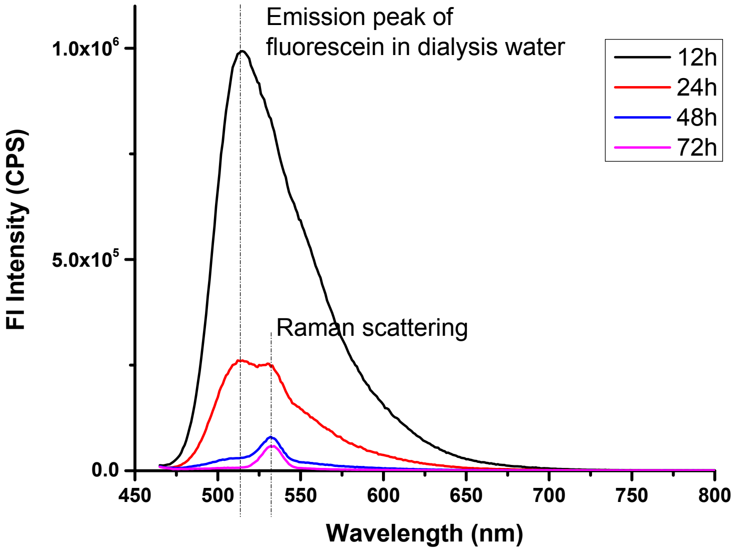

Figure S-1. Intensity of the fluorescence emission peak of fluorescein present in dialysis water decreased during purification every time dialysis water changed. After 3 days of dialysis, there was no fluorescence emission peak in the dialysis water showing the elimination of the free fluorescein in the GluCD-F dispersed in water.

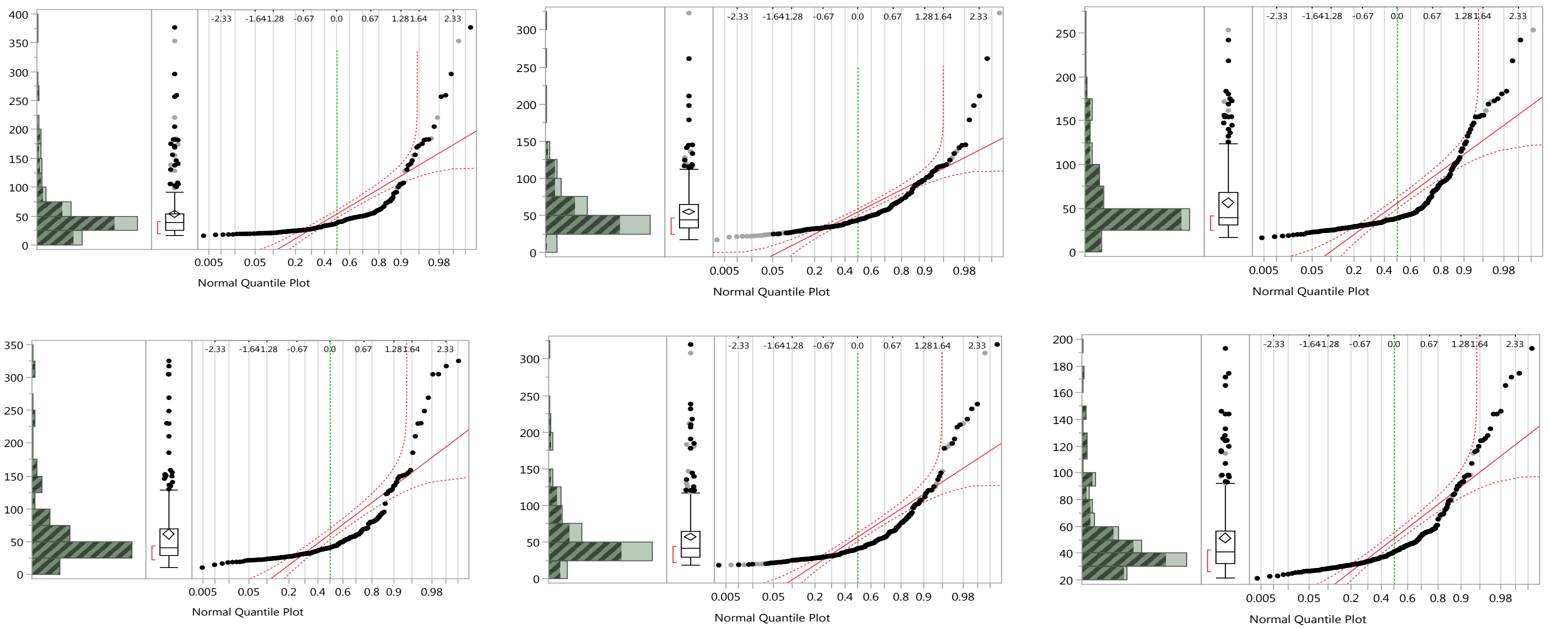

Figure S-2. Area distributions of EBY.VW 5000-C (Hex-) (top three, for three replicates) and EBY.VW 5000-T (Hex-) (bottom three, for three replicates)

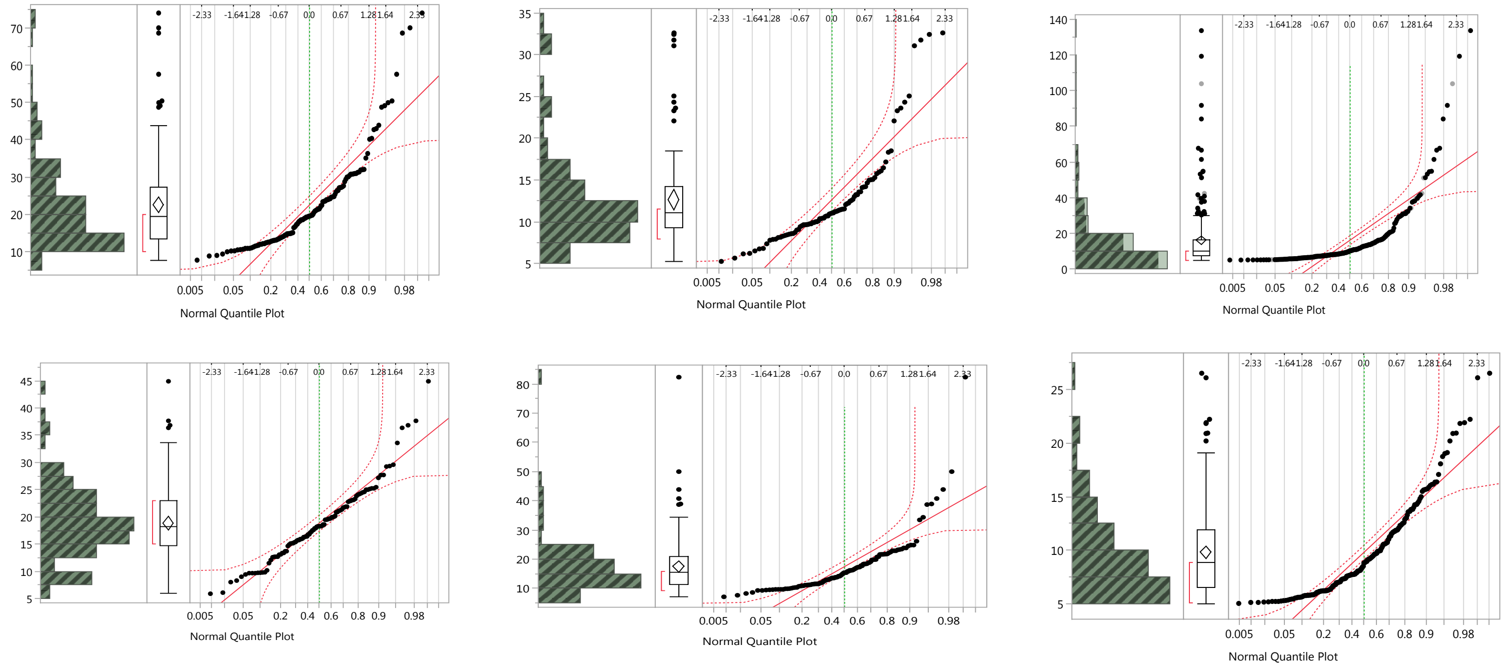

Figure S-3. Area distributions of BC- C (Hex+) (top three, for three replicates) and BC-T (Hex+) (bottom three, for three replicates)

Table S-1. The mean areas of ROIs for each group

	EBY.VW 5000	BC
**Control 1**	62.05	22.55
**Control 2**	54.17	12.67
**Control 3**	54.78	16.20
**Treatment 1**	56.27	18.87
**Treatment 2**	51.36	17.48
**Treatment 3**	57.07	9.83

Table S-2. Comparison between the mean area of ROIs for each group

Level	-Level	p-value
EBY.VW 5000-C	BC-T	<0.0001*
EBY.VW 5000-C	BC-C	<0.0001*
EBY.VW 5000-T	BC-T	<0.0001*
EBY.VW 5000-T	BC-C	<0.0001*
EBY.VW 5000-C	EBY.VW 5000-T	0.5759
BC-C	BC-T	0.6408

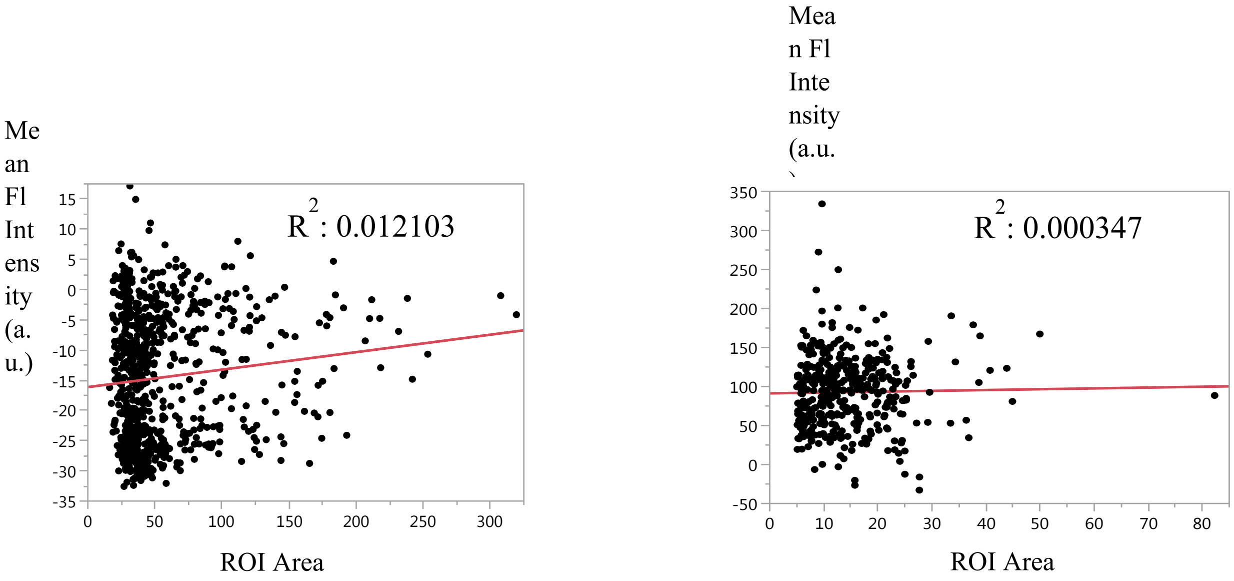

Figure S-4. Bi-variate analysis of Fl intensity vs ROI area for EBY.VW 5000 (Hex-) (left) and BC (Hex+) (right) strains

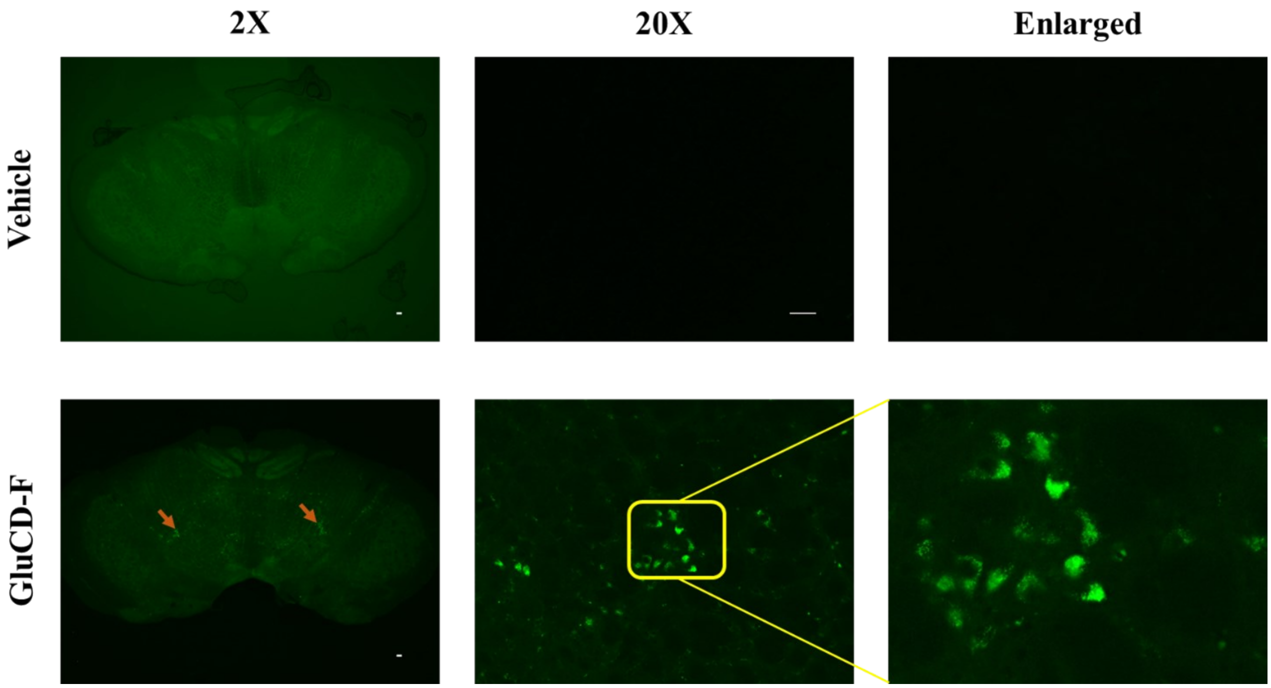

Figure S-5. Representative brainstem fluorescence images from vehicle and glucose carbon dot-fluorescein conjugate (GluCD-F) treated rats. Scale bar (2X): 100 µm, Scale bar (20X): 50 µm
